# Tissue culture-independent approaches to revolutionizing plant transformation and gene editing

**DOI:** 10.1093/hr/uhae292

**Published:** 2024-10-14

**Authors:** Luis Felipe Quiroz, Moman Khan, Nikita Gondalia, Linyi Lai, Peter C McKeown, Galina Brychkova, Charles Spillane

**Affiliations:** Agriculture, Food Systems and Bioeconomy Research Centre, Ryan Institute, University of Galway, University Road, Galway H91 REW4, Ireland; Agriculture, Food Systems and Bioeconomy Research Centre, Ryan Institute, University of Galway, University Road, Galway H91 REW4, Ireland; Agriculture, Food Systems and Bioeconomy Research Centre, Ryan Institute, University of Galway, University Road, Galway H91 REW4, Ireland; Agriculture, Food Systems and Bioeconomy Research Centre, Ryan Institute, University of Galway, University Road, Galway H91 REW4, Ireland; Agriculture, Food Systems and Bioeconomy Research Centre, Ryan Institute, University of Galway, University Road, Galway H91 REW4, Ireland; Agriculture, Food Systems and Bioeconomy Research Centre, Ryan Institute, University of Galway, University Road, Galway H91 REW4, Ireland; Agriculture, Food Systems and Bioeconomy Research Centre, Ryan Institute, University of Galway, University Road, Galway H91 REW4, Ireland

## Abstract

Despite the transformative power of gene editing for crop improvement, its widespread application across species and varieties is limited by the transformation bottleneck that exists for many crops. The genetic transformation of plants is hindered by a general reliance on *in vitro* regeneration through plant tissue culture. Tissue culture requires empirically determined conditions and aseptic techniques, and cannot easily be translated to recalcitrant species and genotypes. Both *Agrobacterium*-mediated and alternative transformation protocols are limited by a dependency on *in vitro* regeneration, which also limits their use by non-experts and hinders research into non-model species such as those of possible novel biopharmaceutical or nutraceutical use, as well as novel ornamental varieties. Hence, there is significant interest in developing tissue culture-independent plant transformation and gene editing approaches that can circumvent the bottlenecks associated with *in vitro* plant regeneration recalcitrance. Compared to tissue culture-based transformations, tissue culture-independent approaches offer advantages such as avoidance of somaclonal variation effects, with more streamlined and expeditious methodological processes. The ease of use, dependability, and accessibility of tissue culture-independent procedures can make them attractive to non-experts, outperforming classic tissue culture-dependent systems. This review explores the diversity of tissue culture-independent transformation approaches and compares them to traditional tissue culture-dependent transformation strategies. We highlight their simplicity and provide examples of recent successful transformations accomplished using these systems. Our review also addresses current limitations and explores future perspectives, highlighting the significance of these techniques for advancing plant research and crop improvement.

## Introduction

The remarkable ability of a single plant cell to regenerate into a whole plant, a characteristic known as totipotency, has allowed the development of innovative plant transformation and gene editing (GE) methods [1, 2]. Recent decades have witnessed significant progress in plant breeding tools, moving from conventional breeding toward molecular engineering and gene transformation [3]. Such advancements have expedited the breeding process, enabling the targeted introduction of specific traits into plants. While traditional plant breeding is limited to combining genes existing within the primary gene pool of a single species, genetic transformation allows access to genes from more distantly related species and even non-plant taxa. Genetic transformation has been transformational in accelerating crop research and the generation of new crop varieties.

In plant transformation protocols, the gene delivery method can be either direct or indirect [4]. While direct gene delivery refers to systems in which the DNA is directly integrated into the cell by physical or chemical methods such as biolistic bombardment or transfection, indirect gene delivery refers to a system in which a biological intermediary is the one mediating the DNA integration in the host cells [4]. *Agrobacterium tumefaciens* (*Agrobacterium*) is the most widely known microorganism used for indirect delivery, due to its natural capacity to efficiently integrate part of its plasmid DNA into plant cells in a cost-effective way [5]. Nevertheless, not only new *Agrobacterium* strains have been developed to improve the efficiency and the range of plant species possible to be transformed by *Agrobacterium*-mediated strategy, but also new bacterial species have been identified as novel indirect gene delivery systems, including for some recalcitrant crops [6].

Despite advancements in traditional plant transformation strategies, challenges persist. Certain plant species and genotypes, especially among monocots, pose difficulties for *Agrobacterium*-mediated transformation [7–9]. *Agrobacterium*-mediated approaches and particularly direct gene delivery strategies can lead to genetic concatemers [10, 11]. Additionally, in major crops, the absence of highly efficient tissue culture regeneration protocols remains a significant barrier, hindering the production of transgenic or edited plants with modified nuclear or plastid genomes [3, 12, 13]. To overcome these issues related to transformation and regeneration efficiency, there is significant research effort to investigate factors affecting callus formation and plant regeneration, particularly through manipulation of plant growth regulators (PGRs). In particular, auxin and cytokinin, as key PGRs, play pivotal roles in regulating the biosynthesis and signaling pathways that govern plant regeneration. The balance between auxin and cytokinin typically determines whether callus develops into shoots or roots [1, 2, 12]. By harnessing developmental regulator (DR) genes involved in the regulation, biosynthesis, and response to PGRs, transformation and regeneration efficiency have been significantly increased in multiple crops. For instance, it has been demonstrated that the ectopic expression of genes encoding for DRs is capable of reprogramming somatic cells and initiating embryogenesis or organogenesis, significantly enhancing transformation efficiency in recalcitrant species and ecotypes [12–14].

Notwithstanding the key role and contribution of tissue culture-dependent (TCD) strategies in transformation and gene editing progress, the simplicity of tissue culture-independent (TCI) strategies, such as floral dip in *Arabidopsis thaliana* (Arabidopsis) [15, 16], arises as cost-effective systems to overcome (a) plant regeneration genotype dependency and (b) the use of specialized equipment and labor associated to plant tissue culture. However, some transformation strategies such as floral dip are not always transferable to crop species. Nevertheless, in recent years, a range of promising TCI strategies applicable to crop breeding have surfaced [3]. This review explores the latest developments in TCI plant transformation and gene editing (Table 1), presenting opportunities and challenges for advancing crop improvement; by harnessing the potential of TCI techniques, plant biotechnology could be further revolutionized, including by contributing to new developments in the synthetic biology era.

**Table 1:** Advantages and disadvantages of TCI plant transformation systems

TCI transformation system			Advantages	Disadvantages	References
Sporophyte transformation and gene editing	Meristem/shoot-based	Meristem injection	1. Simple and fast protocol. 2. No high technical skills required. 3. Low implementation cost. 4. No sterile/aseptic conditions are needed.	1. Low transformation efficiency 2. Lack of replicability 3. High level of chimeric shoots during regeneration	[17–19]
		Cut-dip-budding (CBD)	1. Simple and fast protocol. 2. No high technical skills required. 3. No sterile/aseptic conditions are needed. 4. Rapidly propagated asexually.	1. Requires natural meristem growth through root sprouting, which may not be feasible for all plant species.	[20, 21]
		*De novo* meristem	1. Overcomes genotype dependency barriers. 2. Overcomes the recalcitrancy of crops during plant transformation and regeneration. 3. Low number of plants is required. 4. Low levels of chimerism. 5. Simple and fast protocol.	1. Involves laborious cloning methods for plasmid construction. 2. Selection of appropriate DRs is required. 3. Might require *in vitro* steps (but no tissue culture).	[22]
	Grafting-based		1. Facilitate and reduce the time required to produce CRISPR gene-edited non-transgenic plants.	1. Requires tissue culture for generating transgenic rootstock. 2. Suitability of scion and rootstock needed.	[23]
	Viral-based		1. Simple and fast protocol. 2. No sterile/aseptic conditions are needed. 3. High transformation/gene editing rate.	1. Limited genome inherent capacity of viral genomes. 2. Often requires plants that express Cas protein. 2. Virus infection is often limited to somatic cells (exception exists). 3. The host range is limited for some viruses.	[24–31]
Gametophyte transformation and gene editing	Pollen-based	Pollen tube method	1. Simple and fast protocol. 2. No high technical skills required. 3. Low implementation cost. 4. No sterile/aseptic conditions are needed.	1. Efficiency may vary depending on the plant species. 2. The abundance of pollen may be challenging in some plant species. 3. Requires manual pollination.	[15, 16, 32–45]
		Pollen magnetofection	1. Simple and fast protocol. 2. No high technical skills required. 3. Low implementation cost. 4. No sterile/aseptic conditions are needed. 5. Overcomes the genotype dependency 6. *Agrobacterium* or viruses are not required.	1. Pollen must be at an appropriate developmental stage. 2. The abundance of pollen may be challenging in some plant species. 3. Requires manual pollination.	[46–48]
	Egg cell-based (Floral dip)		1. Simple and fast protocol. 2. No high technical skills required. 3. Low implementation cost. 4. No sterile/aseptic conditions are needed.	1. Limited to specific species or even particular accessions. Flower type, plant size, and the reproductive system are key elements. 2. It is mostly used in Arabidopsis.	[15, 16, 49, 50]

## Sporophyte TCI transformation and gene editing

### Meristem-based tools for TCI transformation and gene editing

**Figure 1:**
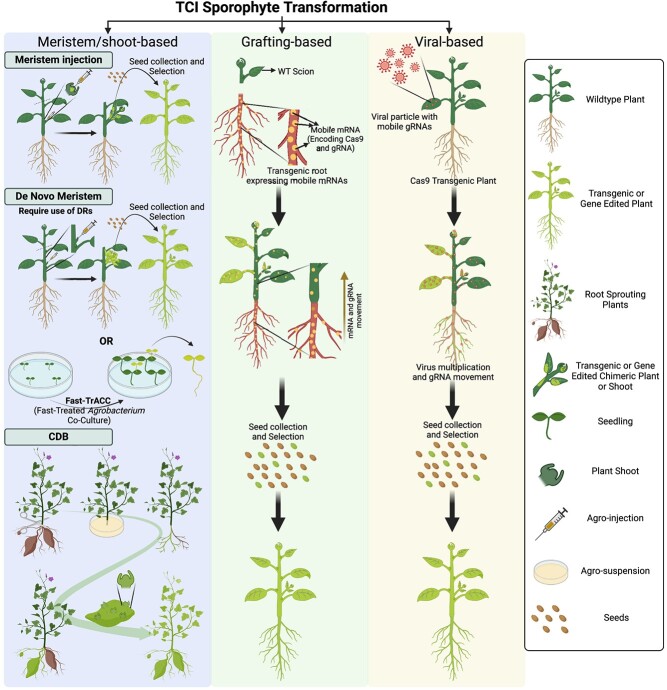
TCI strategies based on sporophyte transformation and gene editing. Schematic representation of transformation and gene editing strategies based on meristem, such as meristem injection, *de novo* meristem induction, and CBD, as well as grafting-based and viral base strategies. DRs: Developmental Regulators; Cas9: CRISPR-associated protein 9; gRNA: guide RNA; mRNA: messenger RNA. Created with BioRender.com.

The growth and development of vascular plant sporophytes is sustained by specialized stem cell niches called meristems (which are typically situated at or near points of apical growth). The shoot apical meristem (SAM) serves as the origin of aerial structures, such as stems, leaves, and flowers, while the root apical meristem (RAM) is the origin of root structures [1, 2, 51–53]. Meristem transformation protocols depend on the insertion of DNA through the infection of plant meristematic regions with *Agrobacterium* [17–19]. Thus, by transforming the meristematic tissue or cells it is possible to generate transgenic or edited shoots without requiring *in vitro* regeneration techniques (Fig. 1). However, despite the apparent simplicity of this transformation methodology, its adoption has been limited by low transformation efficiency, lack of replicability, and high levels of chimerism in the resulting regenerated shoots. In particular, an exceptionally straightforward cut-dip-budding (CDB) delivery system has been developed (Fig. 1). This innovative approach is based on root explant inoculation with *Agrobacterium rhizogenes*, which leads to the regeneration of transgenic roots that subsequently give rise to transformed buds through the natural meristem generation process during root sprouting [20]. Furthermore, the CDB system has been successfully used for TCI transformation and gene editing of tissue culture recalcitrant monocot and dicot succulent plants, by harnessing its capability to regenerate shoots from cut leaves [21].

It is well known that multiple DRs play key roles in the maintenance of meristem identity [12, 51, 52]. As plant cells exhibit remarkable totipotency, displaying the capacity to undergo dedifferentiation or redifferentiation into diverse cell types, this characteristic opens the possibility of boosting meristem differentiation potential by ectopically expressing specific combinations of DRs in somatic cells [12]. Recognizing this, multiple DRs have been used to overcome the problem related to genotype dependency and recalcitrancy in plant regeneration and transformation [9, 12, 13, 54]. While most of the strategies using DRs for plant transformation rely on TCD pipelines, it has recently been demonstrated that *de novo* meristems can be induced in *ex vitro* plants [22]. In particular, using different combinations of genes such as *Zea mays WUSCHEL2* (*ZmWUS2*), *A. thaliana SHOOT MERISTEMLESS* (*AtSTM*) and *A. tumefaciens IPT* (*AtIPT*), it is possible to induce *de novo* meristems to develop from somatic tissue in tobacco, tomato, and grape plants without requiring tissue culture steps [22] (Fig. 1).

### Virus-based strategies for TCI transformation and gene editing

Virus-induced genome editing (VIGE) has emerged as an effective transformation tool, which can enable genome editing. VIGE is considered to be a promising method for obtaining gene-edited plants without tissue culture as viruses can replicate intracellularly and spread intercellularly within their hosts [55]. Compared with traditional methods such as biolistic and *Agrobacterium*-mediated delivery, VIGE can avoid genomic sequence damage as it does not require insertion of the viral genome inside the plant genome, when viral vectors are used based on viruses that do not integrate into the nuclear genome. VIGE relying on clustered regularly interspaced short palindromic repeats (CRISPR) technology usually has two modes of use: one is to deliver guide RNA (gRNA) through viral vectors into pretransformed plants containing a stable expression of endonucleases. Examples include gRNA VIGE systems using tobacco rattle virus (TRV) [26], pea early browning virus (PEBV) [25], and barley stripe mosaic virus (BSMV) [27, 28]. The second option, is to co-deliver gRNA and Cas nucleases into plants through viral vectors, such as the barley yellow striate mosaic virus (BYSMV) [29] and sonchus yellow net rhabdovirus (SYNV) VIGE systems [30]. However, the genomic carrying capacity of viral genomes is inherently limited, making it challenging to deliver relatively large Cas nucleases, suggesting that VIGE technology relying on CRISPR technology has space for further development.

In addition, the infection of viruses is often limited to somatic cells, making it difficult to obtain stable heritable lines arising from editing of meristematic cells. However, certain mobile RNA molecules, like *FLOWERING LOCUS T (FT)* mRNA, are synthesized in the leaves but migrate to the SAM to trigger flowering [56]. Thus, gRNA fused with the Arabidopsis *FT* sequence was integrated into the TRV vector to allow long-distance delivery of gRNA within plants and successfully obtained mutant offspring [31] (Fig. 1). Similarly, after the fusion of 102 bp *FT* mRNA with gRNA, gRNA was successfully introduced into SAM through a cotton leaf crumple virus (CLCrV)-mediated VIGE system, avoiding tissue culture and generating heritable mutant offspring directly [24]. Interestingly, BSMV has been shown to deliver sgRNA into germline or meristematic cells in wheat and barley, though it is dependent on transgenic plants for Cas9 [28, 57, 58]. In contrast, tomato spotted wilt virus (TSWV) has been engineered to successfully deliver both gRNA and Cas9, but it does not transfer the mutation to the next generation [59]. Thus, VIGE is limited by the intrinsic characteristics of viruses, including 1) the host range and tissue specificity; 2) the genome capacity; 3) transmission mode and efficiency; 4) biosafety and biocontainment of engineered viruses [60]. However, VIGE brings new prospects for plant transformation and transgenic applications outside of plant tissue culture.

### Grafting-based transgene-free TCI gene editing

Obtaining stable gene-edited plant lines through CRISPR generally necessitates lengthy tissue culture stages. Additionally, ensuring lines are transgene-free generally requires outcrossing to eliminate CRISPR-associated sequences [61–63]. The need for outcrossing can be overcome by using transient expression, or direct delivery of the Cas9 enzyme and gRNAs into cells, protoplasts, or embryos [64–66]. Although these strategies have been successfully used in various species, the need to regenerate the edited plants via tissue culture still remains.

To address this, it has recently been reported that mobile Cas9 mRNA and gRNA can be generated by fusing them to a mobile tRNA-like sequence (TLS) expressed in a rootstock to which a non-transformed stem is grafted. The mobile Cas9-TLS and gRNA-TLS are able to relocate to the SAMs in the wild-type grafted scion, producing heritable gene-edited lines following the production of seed in flowers derived from the transformed meristems (Fig. 1). This strategy not only produces transgenic-free plant lines, but also removes the need for time-consuming outcrossing related to traditional transgene elimination methods, resulting in a practical approach for diverse breeding programs and crop plants [23]. Of note, even when GE does not require tissue culture, the generation of the transgenic rootstocks is not necessarily TCI, and it will depend on the TCD transformation pipeline of each laboratory. However, it could be possible to use an easy-to-transform genotype or variety as a rootstock for generating elite edited plants in any grafted genotype.

## Gametophyte TCI transformation and gene editing

### Floral dip

**Figure 2:**
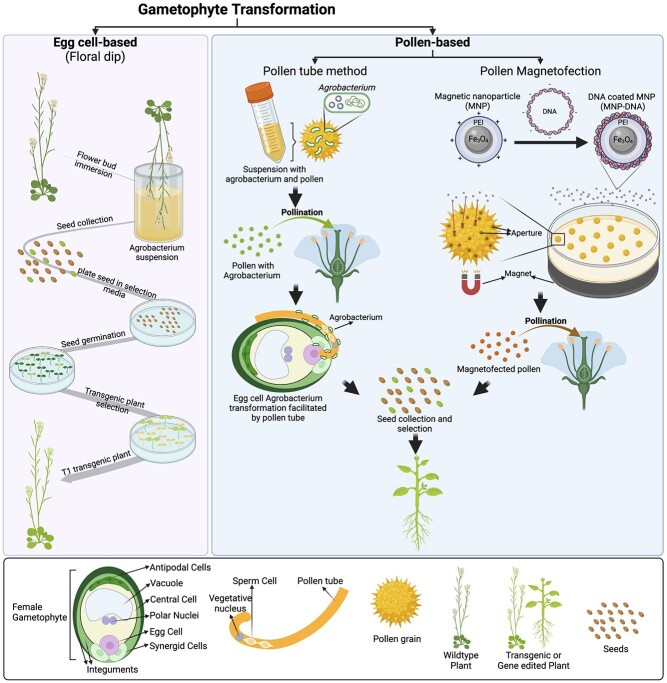
TCI strategies based on gametophyte transformation and gene editing. Schematic representation of egg cell (floral dip) and pollen (pollen tube method and pollen magnetofection) TCI transformation and gene editing strategies. Created with BioRender.com.

The floral dip method stands out as a widely employed and efficient technique in the model plant Arabidopsis [15, 16]. This method has revolutionized the landscape of plant transformation due to its simplicity and effectiveness in introducing foreign genes or transgenes into the Arabidopsis genome. The fundamental steps of the floral dip method involve submerging the flowering Arabidopsis plants into a suspension containing *Agrobacterium* carrying the desired DNA or gene of interest. Thus, facilitating the internalization of *Agrobacterium* into the female gametes [67, 68]. This process is highly efficient, enabling the transformation of many plants within a relatively short period (Fig. 2).

One of the key advantages of the floral dip method lies in its simplicity. The technique eliminates the need for laborious and intricate tissue culture procedures, streamlining the transformation process. Moreover, it allows for the simultaneous transformation of multiple plants, making it particularly useful in large-scale functional genomics studies or when high throughput is required [15, 16]. The method is also cost-effective, as it minimizes the need for specialized equipment and resources. However, it is crucial to acknowledge certain limitations and disadvantages associated with the floral dip method. Its primary applicability is limited to Arabidopsis and a few other plant species, and its efficiency can vary among different accessions [49, 50]. Some of the reasons why the floral dip method may not be suitable for most crop species are: (i) the crop species or variety is recalcitrant to *Agrobacterium*-mediated transformation, (ii) differences in flower structure, (iii) plant physiology and developmental stages, and (iv) scale and throughput difficulties as most crops are generally larger plants.

### Pollen-based strategies for TCI transformation and gene editing

The pollen tube-mediated gene transfer (PTT) technology is a simple strategy that was developed to mimic the natural flowering plant pollination process (Fig. 2). In the course of pollination, pollen falls on the stigma, followed by germination and growth of the pollen tube ultimately leading to two sperm nuclei entering the egg sac (female gametophyte) in order to perform double fertilization [69]. Thus, the opportunity was seen to develop a transformation method by which exogenous DNA could be inserted into the egg cell via a pollen tube. In the absence of a pollen tube, direct insertion of foreign DNA into the egg cell is considered to be very difficult and can easily destroy the egg cell [32]. The PTT methodology was first discovered and successfully applied in cotton [33], and it has been widely applied in many crops in the last two decades, including maize (*Zea mays*) [34, 35], rice (*Oryza sativa*) [36], soybean (*Glycine max L*.) [37, 38], papaya (*Carica papaya*) [39, 40], wheat (*Triticum aestivum* L.) [41, 42], watermelon (*Citrullus lanatus* Thunb.) [43], melon (*Cucumis melo* L.) [44], and peanut (*Arachis hypogaea* L.) [45]. PTT involves three major steps: (i) inserting exogenous genes into pollen tubes, (ii) integration of these genes into the genome of plant, and (iii) transgenic plant selection. This approach avoids the need for complex processes and expensive tools that are required in other transformation methods such as TCD *Agrobacterium*-mediated and biolistic bombardment techniques. Moreover, this non-tissue culture-based methodology can overcome the limitations of genotype specificity [32]. An approach has been reported in peanut involving a combination of PTT and *Agrobacterium*-mediated transformation, which is independent of tissue culture with a frequency of 50% of positive transformants [45].

In the last two decades, gene delivery by nanoparticles has been adapted to GE approaches [47, 70, 71]. However, almost all nanoparticle-based GE tools are dependent on the regeneration of transgenic plants by tissue culture [70, 72, 73]. The magnetic nanoparticle (MNP)-based gene delivery technique, also known as magnetofection, has been proposed as an efficient tool for gene transfer using a magnetic field for the insertion of foreign DNA attached to MNPs [74]. This methodology can directly produce transgenic seeds without tissue culture [47, 74, 75] (Fig. 2). Transformation by pollen has successfully been applied to generate transgenic lines in eudicots such as cotton and pepper, and monocots like lily and maize, with stable inheritance to the next generation observed in both cases [46–48]. It has also been reported that pollen magnetofection methodology is not working well for some monocot species, including sorghum, maize, and lily despite using the same published protocol [76]. This appears to be due to the influence of the pollen operculum (which covers the pollen aperture) in maize, which restricts the exogenous material into the pollen. Thus, it was successfully demonstrated that pollen aperture can be altered and more pollen pores induced by pretreatment of pollen with a transfection buffer at 8°C [48]. Similarly, treatment of maize pollen by ultrasonication to remove the operculum of pollen pore has also been reported [35]. However, this treatment reduced the rate of seed set per ear, mainly due to the reduction of pollen viability due to ultrasonication [35]. It has been hypothesized that the status of pollen from field-grown and plants grown in greenhouses have different ratios of open aperture in pollen, which is mainly due to different environmental stresses such as humidity and temperature, which might affect the opening of pollen pore at certain developmental stages. Thus, it is critical to observe pollen status (for example, by scanning electron microscope (SEM)) before pollen magnetofection, in order to achieve efficient transformation in those monocot species with operculate pollen [48, 77].

## TCI transformation and gene editing in the synthetic biology era

Synthetic biology (SynBio) is an emerging arena of science that seeks to integrate engineering principles into biological systems [78]. In this manner, SynBio not only seeks to develop novel biological parts, systems, or devices but also to redesign existing systems [79, 80]. Particularly, in plant science, SynBio is being promoted as a promising tool for overcoming challenges related to food security, climate change resilience, energy, and the development of new materials and pharmaceuticals, or even space colonization [55, 81, 82].

Many plant transformation techniques apply approaches that could be considered analogous to SynBio, even if they do not use the term, and the application of SynBio design principles could aid the design of more efficient transformation protocols. For instance, the first reported polycistronic system producing multiple gRNA by harnessing the endogenous tRNA processing machinery (tRNA–gRNA system) was reported in plants [83], while previous polycistronic gRNA strategies developed in human cells were based on intercalating 28-nt that were recognized and cut by the co-expressed Csy4 RNase of *Pseudomonas aeruginosa* [84, 85]; this tRNA–gRNA took advantage of the robust, endogenous t-RNA processing machinery without requiring the co-expression of endonucleases [83], creating in this manner a novel and more efficient GE system.

As discussed in previous sections, SynBio strategies based on mobile RNAs [23], engineered viral systems [59, 86], ectopic expression of morphogenic factors [22], and pollen magnetofection [47] have been used for either plant transformation or gene editing without requiring tissue culture steps. While these approaches have been used independently of each other, there is significant potential for developing coordinated or combinatorial systems to improve efficiency. For instance, by combining transient viral-based strategies either with new mobile mRNA systems or with DRs for *de novo* meristem induction. In addition, other plant SynBio technologies, such as tissue-specific knockout (TSKO) or knockdown (TSKD) [87], as well as synthetic genetic circuits [88], could also be integrated into TCI transformation pipelines to control gene expression in different tissues and in this way reprogram multiple biological functions in plants. Furthermore, these TCI-SynBio approaches can also be used together to improve TCI transformation and gene editing efficiency itself.

## Conclusions

Recent advancements in TCI plant transformation and gene editing strategies have not only streamlined methodologies but have also opened new horizons for next-generation plant transformation and gene editing, including for advancing SynBio approaches. While TCI systems have thus far been established and validated in only a limited number of plant species, they hold immense potential for transferability to a broader range of plants and varieties. Where they are not encumbered by intellectual property or GM regulatory ‘freedom to operate’ challenges, TCI strategies have major potential for democratizing, simplifying, and accelerating plant transformation and gene editing (Table 1). Moreover, ongoing developments in both transient and stable plant transformation, coupled with the discovery of new genes influencing shoot induction, cellular division, and other critical stages in plant development, as well as the integration of novel SynBio strategies, collectively herald a promising future for TCI-based transformation and gene editing. However, the pursuit of a unified TCI protocol remains challenging due to differences in plant architecture and life cycles, even among closely related species, whether they are monocots or dicots. Hence, it is crucial to continue developing and improving a wide range of strategies. Overall, the recent and future advances in TCI transformation and gene editing are poised to drive the forefront of plant research and crop improvement, ushering in a new era of possibilities.

## Data Availability

There are no new data associated with this article.
